# Childhood Adversity Is Associated With Increased *KITLG* Methylation in Healthy Individuals but Not in Bipolar Disorder Patients

**DOI:** 10.3389/fpsyt.2018.00743

**Published:** 2019-01-22

**Authors:** Yujie He, Christiaan H. Vinkers, Lotte C. Houtepen, Lot D. de Witte, Marco P. Boks

**Affiliations:** ^1^Brain Center Rudolf Magnus, Department of Psychiatry, University Medical Center Utrecht, Utrecht University, Utrecht, Netherlands; ^2^Brain Center Rudolf Magnus, Department of Translational Neuroscience, University Medical Center Utrecht, Utrecht University, Utrecht, Netherlands; ^3^Department of Psychiatry, Icahn School of Medicine at Mount Sinai, New York City, NY, United States

**Keywords:** DNA methylation, *KITLG*, bipolar disorder, childhood adversity, stress

## Abstract

**Background:** Childhood adversity increases the risk of a range of mental disorders including bipolar disorder, but the underlying mechanisms are still unknown. Previous studies identified DNA methylation levels at the cg27512205 locus on the KIT Ligand (*KITLG*) gene as a mediator between childhood adversity and stress responsivity. This raises the question whether this locus also plays a role in stress related disorders such as bipolar disorder. Therefore, the current study aims to compare the level of *KITLG* (cg27512205) methylation between bipolar patients and healthy individuals and its relation to childhood adversity.

**Methods:** *KITLG* (cg27512205) methylation was measured in 50 bipolar disorder patients and 91 healthy control participants using the HumanMethylation450K BeadChip platform. Childhood adversity in each individual was assessed using the Childhood Trauma Questionnaire. Analyses of the association of *KITLG* methylation with bipolar disorder, the association of childhood adversity with bipolar disorder as well as the association of *KITLG* methylation with childhood adversity in bipolar patients and controls were conducted using linear regression with age, gender, childhood adversity, smoking, and cell-type composition estimates as covariates.

**Results:** *KITLG* (cg27512205) methylation level was significantly lower in bipolar disorder patients (β = −0.351, *t* = −6.316 *p* < 0.001). Childhood adversity levels were significantly higher in the bipolar disorder group (β = 4.903, *t* = 2.99, *p* = 0.003). In the bipolar disorder patients *KITLG* methylation was not associated with childhood adversity (β = 0.004, *t* = 1.039, *p* = 0.304) in contrast to the healthy controls (β = 0.012, *t* = 3.15, *p* = 0.002).

**Conclusions:** *KITLG* methylation was lower in bipolar disorder despite high levels of childhood adversity, whereas childhood adversity was associated with higher *KITLG* methylation in healthy controls. In addition to lower methylation at this locus there is an indication that failure to adjust *KITLG* methylation to high levels of childhood adversity is a risk factor for bipolar disorder.

## Introduction

Bipolar disorder is a severe psychiatric disorder characterized by mood episodes ranging from mania to severe depression (1). The life time prevalence of bipolar disorder is 0.5–1.5% in the general population and 5–10% for first degree relatives (2). Although the pathogenesis of bipolar disorder is not well understood, both genetic and environment factors are involved.

One major detrimental environmental factor for developing mental disorders including bipolar disorder later in life is childhood adversity (3, 4). Childhood adversity encompasses a wild range of adversities before the age of 16, such as physical, emotional and sexual abuse, household poverty, separation from a parent and neglect. Previous studies found that children with childhood adversity have a high risk to develop bipolar disorder (5). However, how childhood adversity contributes to the development of bipolar disorder is still largely unknown.

Recent studies highlight the role of DNA methylation in the pathway of childhood adversity to bipolar disorder (6). DNA methylation is one of the epigenetic mechanisms that can modulate gene expression in response to the environment might account for part of the risk to bipolar disorder (7). Childhood adversity as a detrimental environmental factor could therefore, contribute to DNA methylation differences in key pathways involved in bipolar disorder. In our previous genome-wide DNA methylation analysis, KIT Ligand (*KITLG*) (cg27512205) methylation was positively associated with childhood trauma and served as a mediator between childhood trauma and blunted cortisol stress reactivity in healthy controls (8). Since impaired cortisol stress reactivity is associated with bipolar disorder (9, 10), this could imply an association between *KITLG* methylation with bipolar disorder. Moreover, bipolar disorder patients also report higher levels of childhood adversity (11), which may lead to higher *KITLG* methylation if the previous findings in healthy controls were to be extrapolated to bipolar disorder patients.

Therefore, the current study hypothesizes the presence of higher *KITLG* methylation in bipolar disorder patients as compared to healthy controls in agreement with expected higher level of childhood adversity. To examine this hypothesis, we investigate the relationship between *KITLG* (cg27512205) methylation level in a case-control sample of bipolar disorder patients and healthy controls and the relation to childhood adversity.

## Materials and Methods

### Study Population

Sample recruitment has been previously described (8, 12). In short, 50 bipolar patients and 91 control participants were included at the University Medical Center Utrecht (UMCU). All participants had three or more Dutch grandparents. All participants provided informed consent prior to the inclusion of the study, and the study was approved by the Medical Ethics Committee of the UMCU and performed according to the ICH guidelines for Good Clinical Practice and the latest amendments of the Declaration of Helsinki. All the blood samples from the participants were drawn in the morning before 12 a.m. None of the healthy controls were taking any prescription medication at the time of testing nor did any of the participants ever participate in stress-related research before. To verify drug use, first self-report of current use of psychoactive substances was obtained followed by checking with urine multi-drug screening device (InstantView). If participants smoked daily, they were defined as a smoker. Confirmation of the absence of any mental or physical disorder in the healthy controls was obtained by an independent rater in an interview according to the Mini-International Neuropsychiatric Interview (MINI) plus criteria (13). For bipolar disorder participants only, the Structured Clinical Interview for DSM-IV (SCID) was used to diagnose the clinical characteristics, including mood and psychotic symptoms, number of manic, and depressive episodes, comorbid psychiatric diagnosis and age of disease onset (14). Euthymia in the bipolar disorder patients was established using the Inventory for Depressive Symptoms—Self Report (IDS-SR) (15) and manic symptoms were assessed using the Altman Self-Rating Mania Scale (ASRM) (16). All patients were on a stable (at least 1 month) medication dose. The sample characteristics are provided in Table 1.

**Table 1:** Sample characteristics (*n* = 141).

**Variable n (%) or mean (range)**	**Control**	**Bipolar disorder**	***p***
Number, n	91	50	
Age, years; mean (sd)	33.50 (15.68)	43.52 (12.83)	< 0.001
Female sex, n (%)	44 (48.4%)	25 (50%)	0.853
Smoking, n (%)	11 (12.1%)	18 (36%)	0.001
Age at onset, years; mean (sd)	None	26.37 (11.45)	
Number of episodes; mean (sd)	None	6.39 (5.12)	
Childhood trauma score (mean, sd)	31.77 (8.37)	36.56 (10.28)	0.004
**BIPOLAR DISORDER GROUP**			
Bipolar I, n	None	46	
Bipolar II, n	None	4	
Bipolar disorder Not Otherwise Specified (NOS), n	None	0	

### Childhood Adversity

Childhood adversity was measured using the short version of the Childhood Trauma Questionnaire (CTQ) (17). The Dutch translation of CTQ and validity of the 25 clinical CTQ items has been demonstrated in clinical and population samples (17, 18). One translation item “I believe I was molested” was excluded since this translation was found to be an invalid indicator of childhood sexual abuse in a previous validation study (18). We calculate the sum score of all individual abuse questions to generate a continuous outcome.

### DNA Methylation Analyses

DNA methylation level of *KITLG* (cg27512205) was extracted from previously described Illumina Infinium HumanMethylation450K BeadChip data (12). In short, DNA was obtained from blood using a commercial kit (Qiagen, CA, USA). The DNA concentration and integrity were assessed by riboGreen and BioAnalyser, respectively. Bisulfite conversion was performed by using Zymo Kit (ZYMO Research, CA, USA). Samples were distributed on different chips based on gender and age to reduce batch effects. To remove further systematic differences, the samples were normalized using Beta MIxture Quantile dilation (BMIQ) and batch effects of sentrix array and position were removed with the Combat procedure from the sva package (19). Intensity and quality parameters were obtained from genome studio software. X chromosome, Y chromosome and non-specific binding probes were removed (20). Based on literature (21), probes were excluded based on a detection *P* value > 0.001 and bead count < 5 in 5% of the samples. In addition, probes with SNPs of minor allele frequency >5% within 10 base pairs of the primer were excluded after constructing ancestry estimates as proposed by Barfield et al. (22). 385,882 DNA methylation probes survived quality control, including the *KITLG* cg27512205 probe. All samples were included as none of the samples had more than 1% of probes failed. Cell-type composition estimates were derived using the Houseman procedure (23). Methylation analyses were carried out using M-values (log2 ratio of methylation probe intensity) for better statistical validity (24), but beta values of methylation were used for graphical display.

### Statistical Analysis

Quality control of DNA methylation was conducted with R version 3.1.2 (25). Other statistical analyses were performed using SPSS Statistics 23.0. Analysis of the association of *KITLG* (cg27512205) methylation with bipolar disorder was done using linear regression with *KITLG* methylation as dependent and diagnosis as the main determinant. Age, gender, childhood adversity, smoking, and six different cell-type composition estimates (B cells, CD8 T cells, CD4 T cells, natural killer cells, monocytes, and granulocytes) were included as covariates since they have a potential impact on DNA methylation (26). Differences in childhood adversity between patients and controls were examined using linear regression, in a separate model. This relation was analyzed while adjusted for age, gender and smoking status. The association between *KITLG* methylation and childhood adversity was analyzed by linear regression model in control and bipolar patients separately. Age, gender and smoking were included as covariates.

## Results

### Baseline Characteristics of Bipolar Cohort

A summary of the sample characteristics of bipolar disorder cohort is provided in Table 1. In the bipolar disorder group, 46 participants were diagnosed with bipolar disorder I type and 4 with bipolar disorder II type. The mean age of participants in the control group was significantly lower than in the bipolar disorder (BD) group (control = 33.5, BD = 43.52, *p* < 0.001). The proportion of smokers was significantly higher in the BD group (control = 12.1%, BD = 36%, *P* = 0.001), but no relation was present between *KITLG* methylation and smoking status (β = 0.001, *t* = 0.099, *p* = 0.922). Childhood trauma score was significantly higher in bipolar group than in controls (β = 4.903, *t* = 2.990, *p* = 0.003; model fit: *F* = 8.940, *p* = 0.003, *R*^2^ = 0.060), but these differences were attenuated after adjustment for age gender and smoking(β = 3.043, *t* = 1.817, *p* = 0.071; model fit: *F* = 8.498, *p* < 0.001, *R*^2^ = 0.200). In the bipolar disorder group, comorbid psychiatric diagnosis were: Anxiety disorder Not Otherwise Specified (NOS) (*n* = 1), Generalized anxiety disorder (*n* = 2), Panic disorders (*n* = 4); Agoraphobia without history of panic disorder (*n* = 1), Specific phobia (*n* = 2), Obsessive-compulsive disorder (*n* = 2), Posttraumatic stress disorder (*n* = 1). Considering to the low frequency of the comorbid psychiatric diagnosis in the bipolar disorder group, we do not specifically exam the association of each comorbid psychiatric diagnosis with *KITLG* methylation level.

### KITLG Methylation Analyses

**Figure 1:**
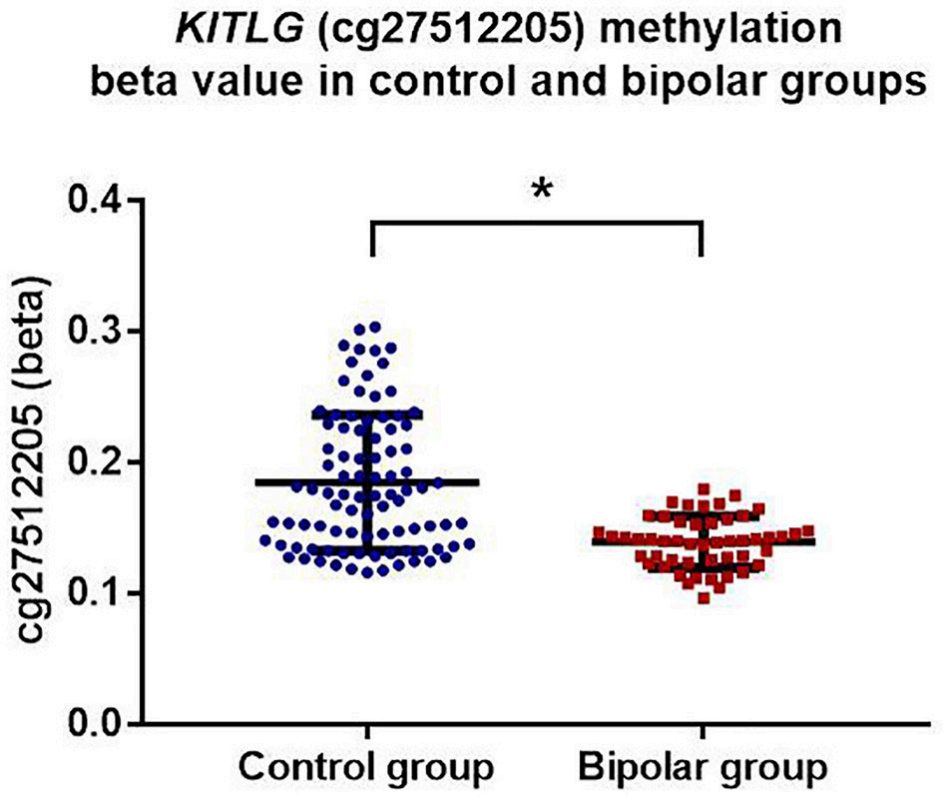
KITLG (cg27512205) methylation (beta value) in healthy controls (blue dots) and bipolar disorder patients (red square). Black bar on each column shows the standard deviation of beta value of KITLG methylation in each group. Mean methylation level of KITLG is significantly lower in the bipolar group (^*^p < 0.001).

*KITLG* methylation level was significantly lower in bipolar disorder patients compared to the healthy controls (mean control = 0.185, mean bipolar = 0.139) (β = −0.351, *t* = −6.316 *p* < 0.001; model fit: *F* = 18.56, *p* < 0.001, *R*^2^ = 0.407) after adjustment for age, gender, childhood adversity, smoking, and cell types. Figure 1 shows the adjusted individual levels of *KITLG* methylation per diagnostic group. No association of medication (mood stabilizer, antidepressant and antipsychotics) with *KITLG* methylation was present in the bipolar disorder group: Mood-stabilizers (β = 0.008, *t* = 1.153, *p* = 0.255); antidepressants (β = 0.006, *t* = 0.732, *p* = 0.468) and antipsychotics (β = −0.008, *t* = −1.279, *p* = 0.208), (Model fit: *F* = 0.937, *p* = 0.488, *R*^2^ = 0.135).

### Association Between Childhood Adversity and KITLG Methylation

**Figure 2:**
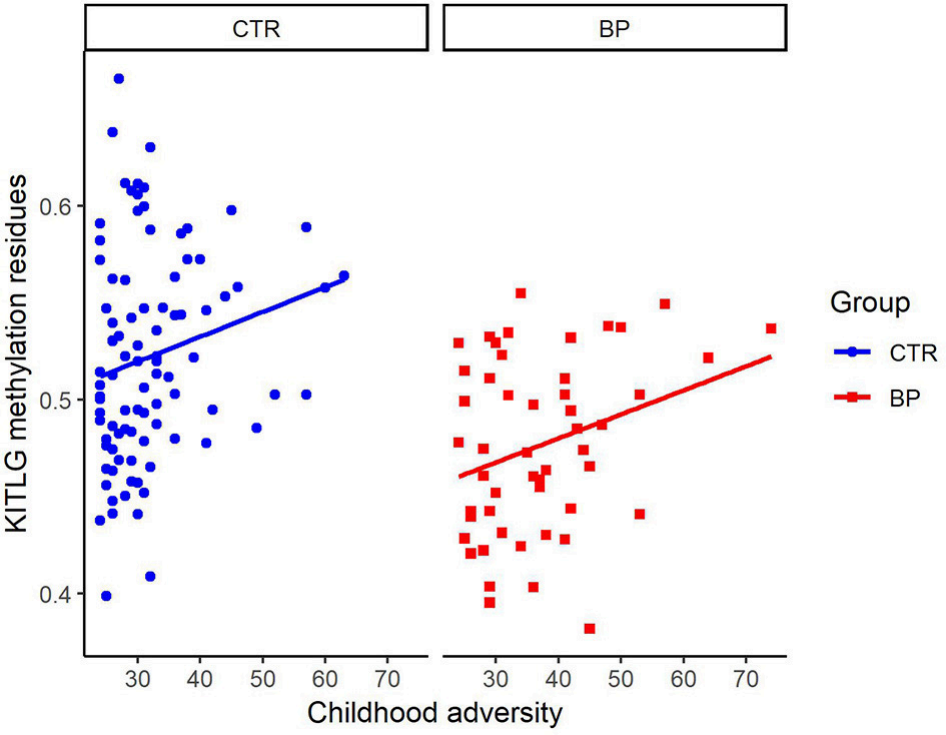
Association of KITLG (cg26512205) methylation level with childhood adversity in both healthy controls (blue dots) and bipolar disorder patients (red square). Y-axis is the beta value of KITLG (cg26512205) methylation level after adjustment for cell type composition, age, gender and smoking. X-axis is the childhood adversity score. Significant positive association between childhood adversity and KITLG methylation was present in the healthy individuals (*b* = 0.012, *p* = 0.002). No significant association between KITLG methylation and childhood adversity in the bipolar disorder patients was present (*b* = 0.004, *t* = 1.039, *p* = 0.304).

Figure 2 shows the association of *KITLG* (cg26512205) methylation level (beta value) with childhood adversity in both healthy controls and bipolar disorder patients. There was no significant association between *KITLG* methylation and childhood adversity in the bipolar disorder patients (β = 0.004, *t* = 1.039, *p* = 0.304), whereas there was a significant positive association between childhood adversity and *KITLG* methylation associated in the healthy individuals (β = 0.012, *p* = 0.002; model fit: *F* = 23.11, *p* < 0.001, *R*^2^ = 0.444).

## Discussion

Here, we follow up the previously reported association of *KITLG* methylation with childhood adversity and stress reactivity by exploring the relationship between *KITLG* DNA methylation levels at the locus cg27512205 and bipolar disorder. To our knowledge, this is the first study to report the association of *KITLG* methylation with bipolar disorder. We found lower DNA methylation levels at this stress related gene in bipolar disorder patients (*n* = 50) than in healthy controls (*n* = 91). In contrast to the positive association between childhood adversity with *KITLG* methylation in controls, we did not observe such an association in bipolar disorder patients. These findings suggest that failure to increase *KITLG* methylation in response to childhood adversity may constitute a risk factor for bipolar disorder.

Previously, we already reported of the positive association between *KITLG* methylation and childhood adversity in healthy controls (8). It is this finding that led to the expectation of *KITLG* hypermethylation among bipolar disorder patients exposed to higher levels of childhood adversity. However, the current study found *KITLG* hypomethylation in bipolar disorder patients and no relationship between childhood adversity and *KITLG* methylation in this group. This finding is consistent with a model whereby *KITLG* hypermethylation after childhood adversity is adaptive and failure to adapt is a characteristic of bipolar disorder patients. However, visual inspection of the relations between childhood adversity and *KITLG* methylation (Figure 2) points to systematic lower *KITLG* methylation in bipolar disorder.

Although unexpected, these findings are consistent with other recent reports that the protein coded by *KITLG* gene, known as stem cell factor (SCF), is significantly higher in children of bipolar disorder patients who develop mood disorder later in life (27). These higher levels of the *KITLG* protein SCF before disease onset are consistent with less repression on gene expression and transcription (28) and DNA hypomethylation at this locus. The specific *KITLG* locus (cg27512205, chr12: 88579621) that we focused on in the current study, is located in a H3K27ac-enriched region as well as on the 5' end of a CpG island near the *KITLG* gene. Mechanistically, DNA hypomethylation in the H3K27ac-enriched region is associated with a more open chromatin structure which indicates active gene transcription (29, 30). Moreover, DNA methylation differences frequently occur in CpG island shores and subsequently affect gene transcription and expression (31). These two co-occurrences suggest that *KITLG* hypomethylation at this CpG locus could indeed alter gene transcription and SCF levels. Another factor that could influence gene transcription level are genetic variants. For instance, gene polymorphism of *FKBP5*, an important functional regulator of the glucocorticoid receptor (GR), can mediated gene–childhood trauma interactions through DNA methylation level (32) and similarly genetic variants modify the methylation response to maternal famine (33). The *KITLG* locus in the current study contains just one genetic variant with no functional relevance for expression and therefore no indication of a role in genetic regulation is currently available.

A putative link between *KITLG* function and bipolar disorder is that the ligand of the C-kit receptor (SCF), is involved in hematopoiesis (34), neurogenesis, and neuroprotection (35) and induces glucocorticoid receptor gene (*NR3C1*) expression in response to stress induced erythropoiesis (36). This implies a positive regulation of *KITLG* gene to *NR3C1* expression, a key gene in the stress response (37, 38), that in term plays a role in bipolar disorder (9) and the response to trauma (39–41). Though the current finding is based on blood, the database from Hannon et al, shows that methylation of this specific *KITLG* locus (cg27512205) in the blood is significantly correlated with prefrontal cortex and superior temporal gyrus in the brain (42). This implies that blood *KITLG* methylation may serve as a proxy for *KITLG* methylation in these brain areas.

Some limitations need to be considered when interpreting these results. First, the focus on one specific locus (cg27512205) based on our previous work, could potentially neglect DNA methylation at other genes that play a role in bipolar disorder. Using available Illumina Infinium HumanMethylation450K BeadChip data, an unbiased genome-wide DNA methylation analysis to investigate the interaction between bipolar disorder and childhood adversity may further our understanding of epigenetic difference related to childhood adversity and bipolar disorder. Second, though for some epigenetic loci blood may provide a reasonable proxy based on concordances in methylation patters between blood and brain (43, 44), it is a limitation considering that bipolar disorder is a psychiatry disorder residing largely in the brain. Another limitation of the study is that the Illumina 450 k BeadChip cannot distinguish between 5-Methylcytosine and 5-Hydroxymethylcytosin.

In conclusion, this study shows that *KITLG* methylation level is significantly lower in bipolar disorder despite relatively high childhood adversity exposure in bipolar disorder patients. This suggests a failure to adjust this epigenetic mark in response to childhood adversity in those vulnerable to bipolar disorder.

## Ethics Statement

All subjects gave written informed consent before participation. This study was carried out in accordance with GCP guidelines and was approved by the Medical Ethics Committee of the UMCU.

## Author Contributions

MB and CV designed the study. LdW, MB, and CV collected the data. MB, LH, and YH performed statistical analysis. MB, CV, and YH wrote the manuscript. All authors read and approved the final manuscript.

### Conflict of Interest Statement

The authors declare that the research was conducted in the absence of any commercial or financial relationships that could be construed as a potential conflict of interest.
